# Chondrocyte sheet *in vivo* cartilage regeneration technique using miR-193b-3p to target MMP16

**DOI:** 10.18632/aging.102237

**Published:** 2019-09-06

**Authors:** Xia Chen, Ruhong Zhang, Qun Zhang, Zhicheng Xu, Feng Xu, Datao Li, Yiyuan Li

**Affiliations:** 1 Department of Plastic and Reconstructive Surgery, Shanghai Ninth People’s Hospital, Shanghai Jiao Tong University School of Medicine, Shanghai 200011, China

**Keywords:** cartilage regeneration, cell sheet, extracellular matrix (ECM), tissue engineering, miR-193b-3p

## Abstract

Stable cartilage regeneration has always been a challenge in both tissue engineering research and clinical practice. This study explored the feasibility of using a chondrocyte sheet technique stimulated by microRNAs to regenerate cartilage. We tested the involvement of hsa-miR-193b-3p in the microtia patient remnant auricular chondrocyte extracellular matrix (ECM). We observed *in vitro* chondrocyte proliferation, ECM synthesis, as well as the increase in the expression of type II collagen (COL2A1) and decrease in the expression of matrix metalloproteinase 16 (MMP16) of the chondrocyte sheets. COL2A1 deposition and MMP16 degradation of regenerative cartilage tissue were examined *in vivo*. A dual-luciferase reporter showed that the MMP16 gene was the direct target of miR-193b-3p. These results suggested that miR-193b-3p promotes chondrocyte sheet ECM synthesis by inhibiting MMP16. Since the evidence suggests that MMP16 is a critical regulator of chondrocyte ECM, this finding points the way towards a method that both strengthens the ECM and inhibits MMPs.

## INTRODUCTION

Cartilage repair and reconstruction is difficult because cartilage is avascular and has a limited nutrition metabolism, and also because it is inherently difficult to regenerate tissue. One tissue engineering approach that has been used recently is a scaffold material-free cell sheet technique. Cell sheets have numerous advantages: they are autologous, exclude foreign materials, do not cause inflammation and eliminate the risk of an immune rejection [1].

The extracellular matrix is a key component of cell sheets. The ECM is subject to both synthesis and degradation; promoting synthesis while suppressing degradation is crucial. Human auricular cartilage matrix is rich in COL2A1, consistent with elastic cartilage [2, 3]. However, matrix metalloproteinases (MMPs), which belong to the protease superfamily, are capable of degrading all kinds of extracellular matrix proteins [3–6]. Ideally, a method can be found that both strengthens the ECM and inhibits MMPs.

MicroRNAs (miRNAs), a type of small endogenous noncoding RNA with wide-ranging regulatory effects, are implicated in cell proliferation [7], apoptosis [8, 9], differentiation [7, 10] and ECM metabolism [11–15]. Previous studies have revealed that miR-193b-3p may regulate chondrogenesis, chondrocyte metabolism [16, 17] and matrix metalloproteinase in osteoarthritis [18]. MiRNAs suppress target gene expression by binding to the 3’untranslated region (3’UTR), either preventing mRNA translation or triggering RNA degradation [19, 20]. Furthermore, some research work has detected relationships between MMPs and miRNAs [21–23]. Using a bioinformatic approach to predict the miRNA target gene, we found that MMP16 is in all likelihood a critical regulator of chondrocyte ECM. MMP16 is a membrane-type metalloprotease which directly degrades some matrix molecules [24].

The functional role of miR-193b-3p in auricular chondrocyte extracellular matrix synthesis and its control and regulation mechanisms remain unknown. Our study sought to assess the effect of miR-193b-3p on auricular chondrocyte ECM synthesis and elucidate the underlying action mechanism. To do this, we investigated the expression levels of COL2A1, as well as the levels of MMP16.

## RESULTS

### Transfection efficiency and chondrocyte proliferation

**Figure 1:**
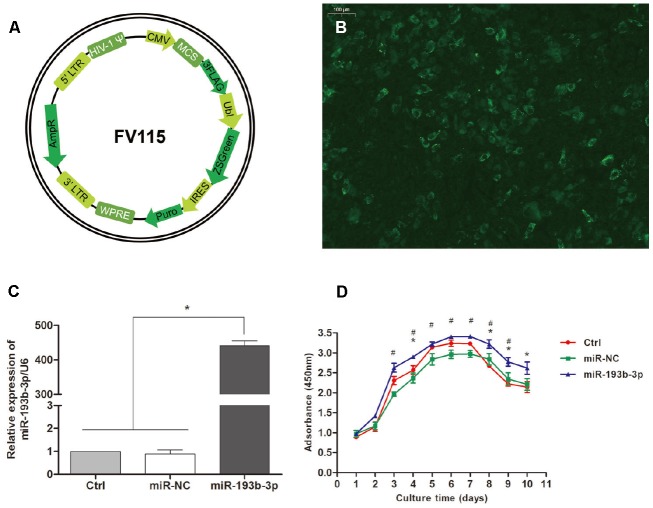
**Transfection efficiency and chondrocyte proliferation.** (**A**) Recombinational lentivirus construction. (**B**) Immunofluorescence staining showing the cells successfully transfected with lentiviruses (magnification x100; bar 100 μm). (**C**) The relative expression of miR-193b-3p/U6 in the experimental group was much higher (p < 0.05) (n=3). (**D**) Proliferation of the chondrocytes cultured in different groups (p < 0.05, * = difference between miR-193b-3p and control groups, ^#^ = difference between miR-193b-3p and miR-NC groups) (n=3).

Figure 1B and 1C shows that human auricular chondrocytes were successfully transfected with miR-193b-3p; the relative expression of miR-193b-3p/U6 in the experimental group was 434.97 fold (p = 0.0125). Figure 1D shows the proliferation of the chondrocytes cultured in different groups. In the medium supplemented with miR-193b-3p mimics, cell proliferation was superior to the blank control and mimics-NC groups. In addition, cell proliferation in the miR-193b-3p mimics-NC medium was very similar to the blank control group.

### *In vitro* characterizations of chondrocyte sheets

**Figure 2:**
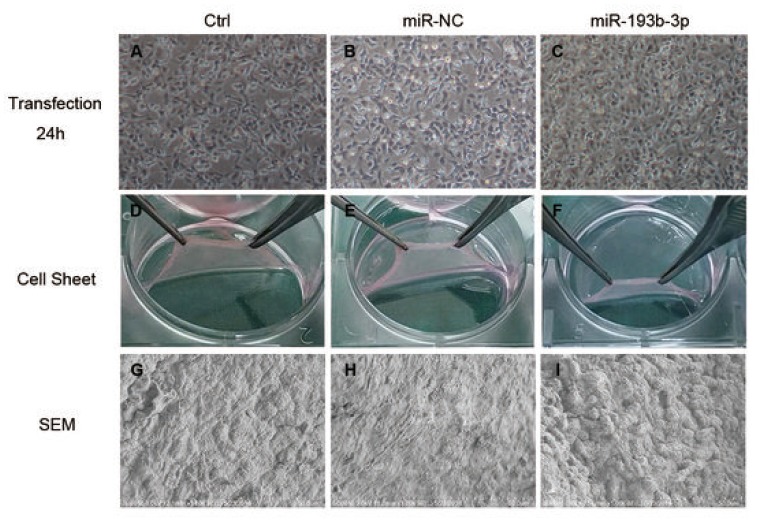
***In vitro* characterization of chondrocyte sheets (n=3).** (**A**–**C**) Chondrocytes transfected for 24 hours in different groups (magnification x40). (**D**–**F**) The chondrocyte sheets formed after 8 weeks could be detached easily and completely. (**G**–**I**) SEM detection of chondrocyte sheets in different groups (magnification x1000; bar 50 μm).

Figure 2A–2C show chondrocytes transfected for 24 hours in different groups. There was no discernible difference among these three groups. Figure 2D–2F show the chondrocyte sheets formed after 8 weeks which could be easily and completely detached from the plate bottom with a pair of tweezers. The cell sheets in the miR-193b-3p group were the thickest and most tenacious; the quality of the cell sheets in the blank control and miR-NC groups was inferior. In the miR-NC group, it was easier to shrink the cell sheet rims while detaching them. These results accorded with SEM detection, which showed an abundance of overlaying cells. Chondrocyte sheets revealed a dense cell network – a mass of ECM secreted by the cells – with tight cell junctions. The surface was wave-like in the miR-193b-3p group (Figure 2I), but smooth in the miR-NC group (Figure 2H), with intermediary results for the blank group (Figure 2G).

### *In vitro* extracellular matrix synthesis of the chondrocyte sheets

**Figure 3:**
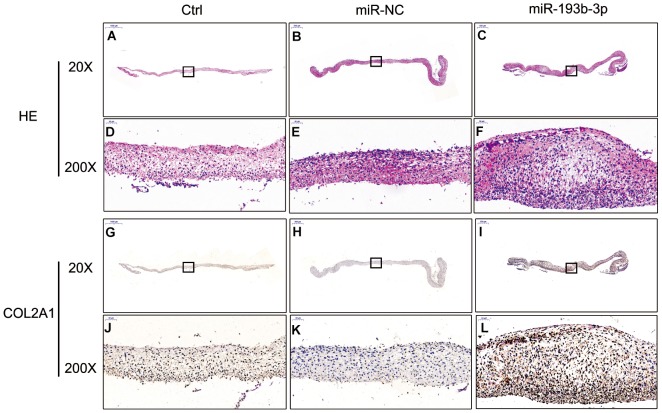
***In vitro* H&E staining and immunohistochemical assay of the chondrocyte sheets in different groups (n=3).** (**A**–**C**) H&E staining magnification 20 times (bar 500 μm). (**D**–**F**) Zoom square magnification x200; bar 50 μm. (**G**–**I**) Immunohistochemical assay magnification 20 times (bar 500 μm). (**J**–**L**) Zoom square magnification x200; bar 50 μm.

**Figure 4:**
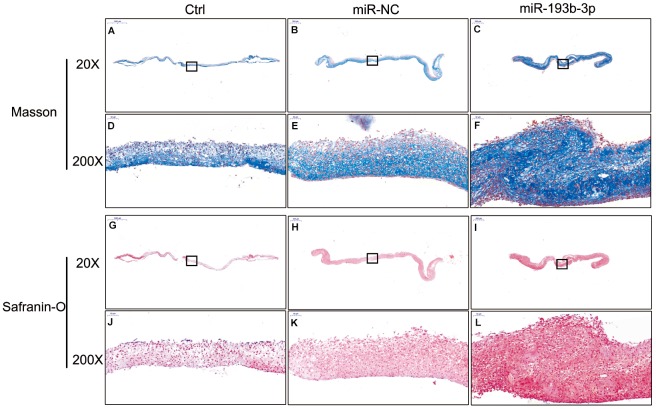
***In vitro* masson and safranin-O staining of the chondrocyte sheets in different groups (n=3).** (**A**–**C**) Masson staining magnification 20 times (bar 500 μm). (**D**–**F**) Zoom square magnification x200; bar 50 μm. (**G**–**I**) Safranin-O staining magnification 20 times (bar 500 μm). (**J**–**L**) Zoom square magnification x200; bar 50 μm.

H&E staining and immunohistochemical assay (Figure 3) show that chondrocyte quantities and COL2A1 synthesis in the miR-193b-3p were higher than in the other groups. Masson and safranin-O staining (Figure 4) show that chondrocyte quantities and extracellular matrix collagen synthesis were higher in the miR-193b-3p than in the other groups. The histological and immunohistological results in the miR-NC treated group were similar to the blank control group. The larger amounts of chondrocyte proliferation and COL2A1 deposited in the miR-193b-3p group than in the control and miR-NC groups indicated that miR-193b-3p significantly promoted the synthesis of the chondrocyte extracellular matrix.

**Figure 5:**
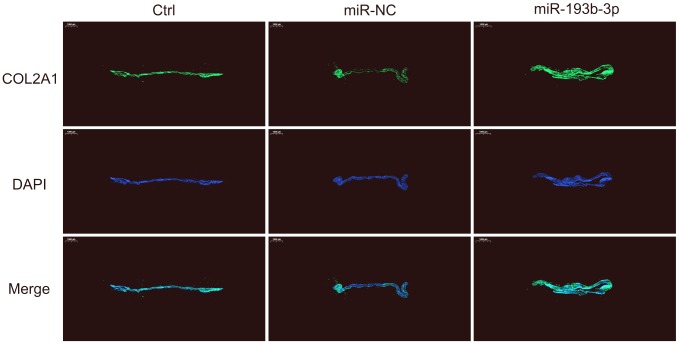
***In vitro* COL2A1 immunofluorescence staining of the chondrocyte sheets in different groups.** Fluorescence microscopy imaging (bar 1000 μm) of the nucleus (blue) and COL2A1 (green) (n=3).

We also used immunofluorescence staining to detect the COL2A1 deposits of chondrocyte sheets in different groups (Figure 5). Fluorescence microscopy imaging of the nucleus (blue) and COL2A1 (green) was employed. It showed that the expression level of COL2A1 treated with miR-193b-3p was higher than the other two groups, which is consistent with the immunohistochemical results.

### *In vivo* cartilage regeneration of the cell sheets

**Figure 6:**
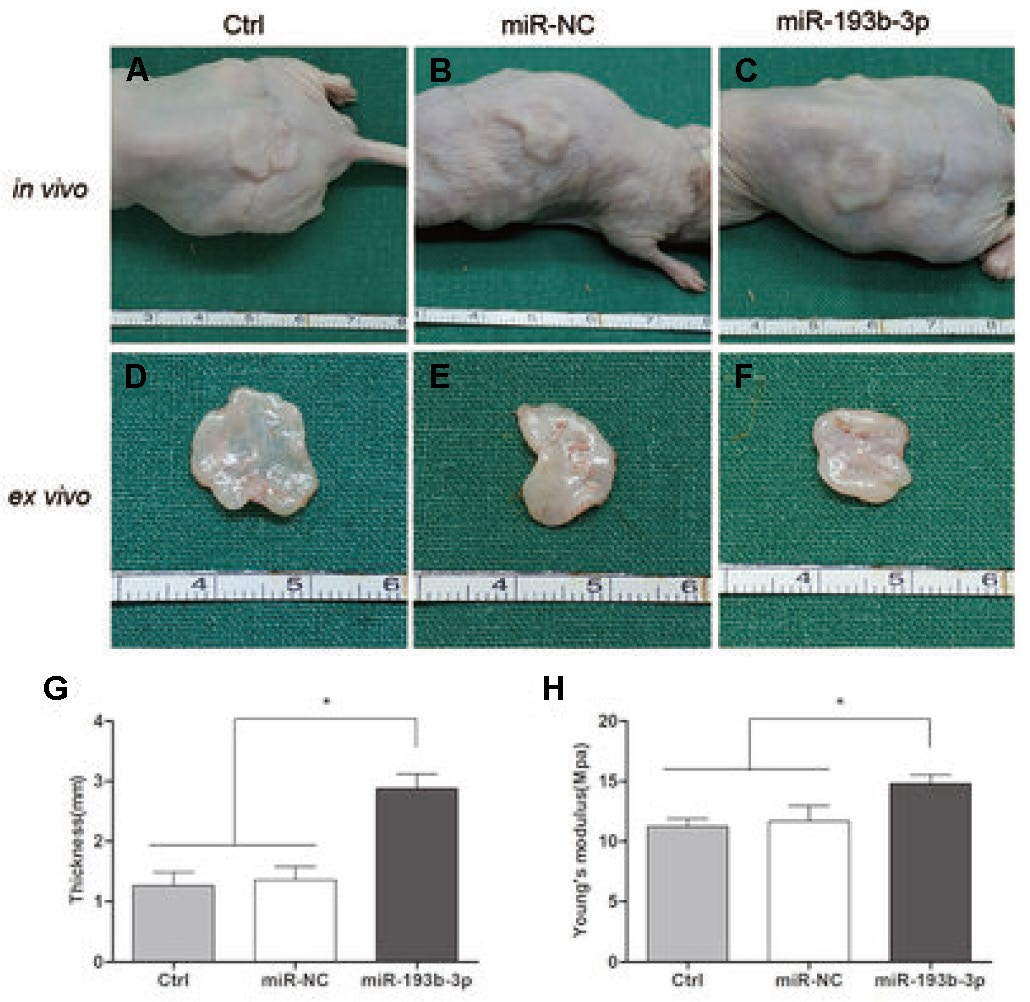
***In vivo* cartilage regeneration of the cell sheets after 8 weeks.** (**A**–**C**) Subcutaneous gross view of cell sheets in nude mice after eight weeks of transplantation. (**D**–**F**) Gross view of cartilage tissue *ex vivo* (**G**) Thickness of cartilage tissue. (**H**) Young’s modulus of cartilage tissue (n=3).

Eight weeks after subcutaneous transplantation of the cell sheets in nude mice, the implants were analyzed by histological, immunohistochemical and immunofluorescence assays. The gross view clearly revealed that white, glossy neocartilage tissue had formed *in vivo* in all three groups (Figure 6). Tissue treated with miR-193b-3p appeared to be the thickest (p = 0.0003) and most solid (p = 0.0293) of all three groups.

**Figure 7:**
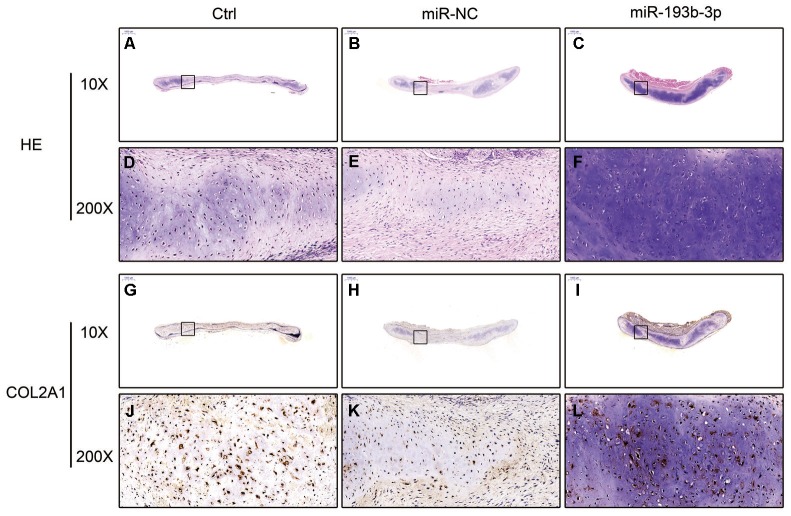
***In vivo* regenerative cartilage H&E staining and immunohistochemical assay in different groups (n=3).** (**A**–**C**) H&E staining magnification 10 times (bar 1000 μm). (**D**–**F**) Zoom square magnification x200; bar 50 μm. (**G**–**I**) Immunohistochemical assay magnification 10 times (bar 1000 μm). (**J**–**L**) Zoom square magnification x200; bar 50 μm.

**Figure 8:**
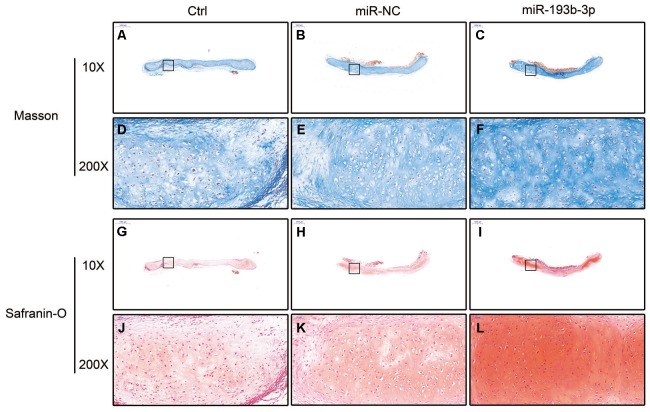
***In vivo* regenerative cartilage masson and safranin-O staining in different groups (n=3).** (**A**–**C**) Masson staining magnification 10 times (bar 1000 μm). (**D**–**F**) Zoom square magnification x200; bar 50 μm. (**G**–**I**) Safranin-O staining magnification 10 times (bar 1000 μm). (**J**–**L**) Zoom square magnification x200; bar 50 μm.

In the histological examination, chondrocyte sheets developed into mature cartilage tissue with typical lacuna structures. There were many more lacunae in the miR-193b-3p group than in the blank control and miR-NC groups. The immunohistochemical assay also confirmed a strong positive COL2A1 result in the experimental group, which was more deeply stained and contained larger COL2A1 deposits than the other groups (Figure 7). Masson and safranin-O staining (Figure 8) showed that there was deeper stained and higher extracellular matrix collagen synthesis in the miR-193b-3p than in the other groups.

**Figure 9:**
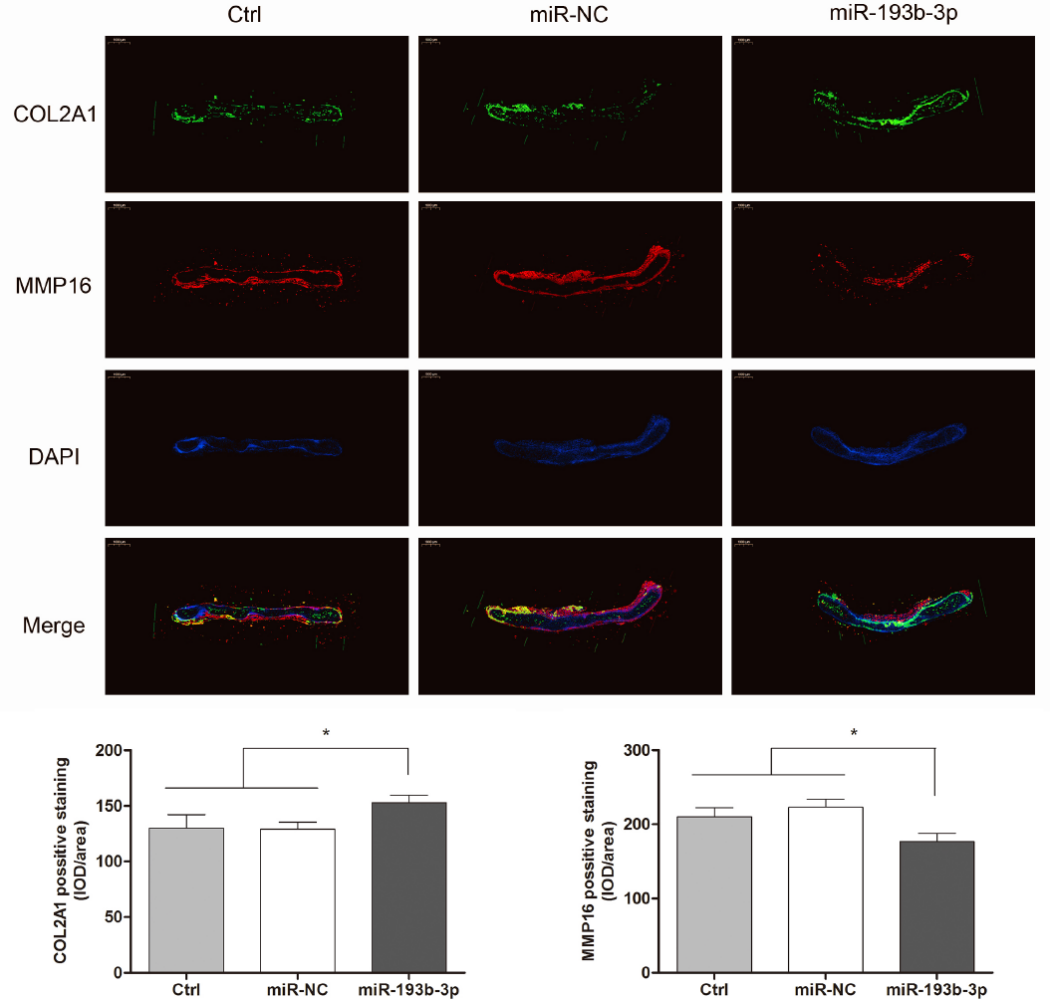
***In vitro* regenerative cartilage COL2A1 and MMP16 co-localization immunofluorescence staining in different groups (n=3).** Fluorescence microscopy imaging (bar 1000 μm) of the nucleus (blue), COL2A1 (green) and MMP16 (red).

COL2A1 and MMP16 expression of cartilage tissue *in vivo* in different groups was observed with immunofluorescence assay (Figure 9). Fluorescence microscopy imaging of the nucleus (blue), COL2A1 (green) and MMP16 (red) was employed. Compared with the control and miR-NC groups, the COL2A1 expression in the miR-193b-3p group showed a significant increase and MMP16 expression was the lowest. These results suggested that miR-193b-3p positively impacts synthesis of the auricular chondrocyte extracellular matrix.

### Mechanism: effect of miR-193b-3p’s target gene MMP16 on chondrocyte sheets

**Figure 10:**
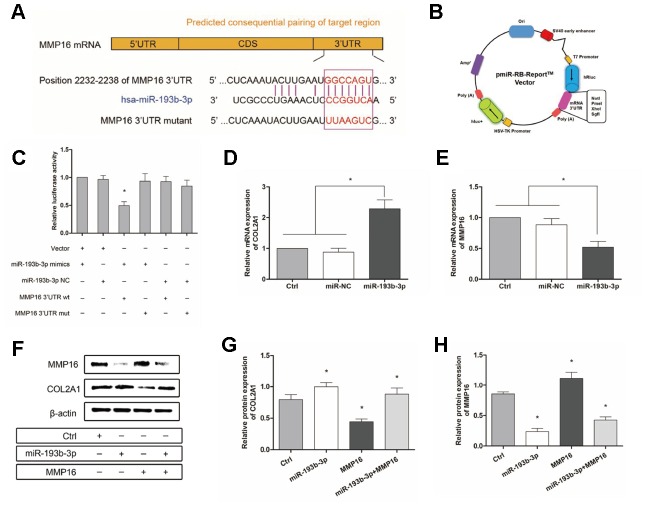
**Mechanism: effect of miR-193b-3p’s target gene MMP16 on chondrocyte sheets.** (**A**) Sequence of the predicted miR-193b-3p binding site with the 3’UTR of MMP16 (position 2232-2238). (**B**) Vectors used in dual-luciferase reporter assay containing the seed sequence of MMP16 3’UTR. (**C**) Reduced relative luciferase activity transfected with MMP16 and miR-193b-3p, compared with the negative control or MMP16 mutant (p < 0.05) (n=3). (**D**, **E**) Gene expression of COL2A1 and MMP16 (p < 0.05) (n=3). (**F**) Protein expression of COL2A1 and MMP16 for chondrocytes cultured in different groups. (**G**, **H**) Quantitative protein level of COL2A1 and MMP16 in different groups (p < 0.05) (n=3).

To gain further insight into the mechanism by which miR-193b-3p regulates the synthesis of chondrocyte ECM, we used the software program TargetScanHuman 7.2 (updated March 2018) to predict the potential targets of miR-193b-3p. Among the candidate target genes, MMP16 (position 2232-2238 of MMP16 3’UTR) (Figure 10A) attracted our attention. We performed luciferase reporter assay to verify whether MMP16 was the target gene of miR-193b-3p. Vectors containing the MMP16 3’UTR seed sequence in the predicted consequential pairing of target region, interacted with miR-193b-3p (Figure 10B). The lower relative luciferase activity in the transfection with MMP16 and miR-193b-3p, compared with the negative control or MMP16 mutant (p = 0.0153) (Figure 10C), supported our hypothesis. In the present study, dual-luciferase reporter assay proved that MMP16 was a target gene of miR-193b-3p.

**Figure 11:**
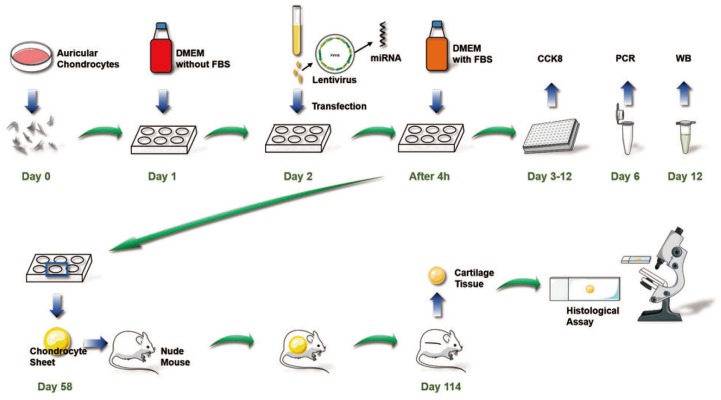
**Overall chondrocyte sheet tissue engineering process to promote cartilage regeneration *in vivo* used in this study.**

Subsequently, Figure 10D and 10E show the gene expression of COL2A1 and MMP16 (p < 0.0001 and p = 0.0125 respectively), while Figure 10F shows the protein expression for chondrocytes cultured in different groups. There was a statistically significant increase in both the COL2A1 gene and protein levels and a decrease in MMP16 levels following treatment with miR-193b-3p compared with the control or miR-NC groups (Figure 10G and 10H). The quantitative protein level of MMP16 also demonstrated that miR-193b-3p + MMP16 overexpression was repressed and COL2A1 promoted. Furthermore, quantitative PCR and western blot also demonstrated that miR-193b-3p can inhibit MMP16 expression in our study. All these results confirm that miR-193b-3p can promote synthesis and accumulation of ECM by regulating the expression of target gene MMP16.

## DISCUSSION

Cell sheet technology is now widely used in the field of tissue engineering and regenerative medicine [1, 25–27]. The cell sheet is composed of cells and extracellular matrices. The ECM in cell sheets provides a nutritive, structural microenvironment for cell adhesion and growth, as well as promoting tissue regeneration *in vivo* [28]. Cell sheets can adhere tightly to host tissues or each other to constitute abundant ECM in cell sheets. Previous studies reported that bone marrow stromal cells (BMSCs) first differentiate into chondrocytes, then form cell sheets to regenerate cartilage due to the limitation of chondrocyte numbers [29, 30]. There is a risk of chondrosteosis with this approach. In our study, we used chondrocyte sheets to regenerate cartilage, thereby promoting chondrocyte proliferation, but also enhancing ECM synthesis.

MiRNAs play an important role in cartilage regenerative medicine [31, 32]. It has been reported that MiR-193b-3p promotes hMSC chondrogenesis and metabolism in chondrocytes [15]. However, whether miR-193b-3p is involved in the synthesis of human auricular chondrocyte ECM and cartilage regeneration remains unknown. To investigate this possibility, the expression of miR-193b-3p in auricular chondrocytes was up-regulated via miRNA transfection, which was observed to increase auricular chondrocyte proliferation, accompanied by augmented gene and protein expression of COL2A1 and decrease the expression levels of MMP16. The neocartilage tissue thickness and mechanical strength measurement results show that miR-193b-3p promoted chondrocyte ECM synthesis. In this study, *in vivo* neocartilage tissue thickness was uneven. In general, the peripheral region was thicker than the central part. We measured the thickness of the central part so as to provide the same measurement for each sample, and also because the central part is the key, weak neocartilage region. Tissue treated with miR-193b-3p appeared to be the thickest and most solid of all three groups. In addition, the histological, immunohistochemical and immunofluorescence *in vitro* and *in vivo* results also demonstrated higher COL2A1 deposition and less MMP16 degradation in the miR-193b-3p group compared with other groups. These results demonstrate that miR-193b-3p is a positive regulator of chondrocyte ECM synthesis in cartilage regeneration.

The chondrocyte sheet tissue engineering processes used in this study to promote cartilage regeneration *in vivo* are shown in Figure 9. We are the first researchers to describe this miR-193b-3p-mediated cartilage regeneration technique using chondrocyte sheets to promote auricular chondrocyte extracellular matrix synthesis by regulating the target gene MMP16.

To further understand the mechanism of miR-193b-3p in auricular chondrocyte ECM, the present study predicted and verified the target gene MMP16. Luciferase reporter assay, qPCR and western blot analysis results confirmed that MMP16 was the target gene of miR-193b-3p. In order to remove uncertainty concerning the functional role of MMP16 modulated by miR-193b-3p, a rescue procedure was performed using MMP16 overexpression. Again, the results supported the initial hypothesis: miR-193b-3p, which targets MMP16, is a key molecule in cartilage regeneration. The results showed that miR-193b-3p repressed the key target gene MMP16 and stimulated the synthesis of the chondrocyte extracellular matrix. In addition, we describe in detail, for the first time, the mechanism by which hsa-miR-193b-3p promotes auricular chondrocyte ECM synthesis.

## MATERIALS AND METHODS

### *In vitro* chondrocyte isolation, culture and chondrocyte sheet engineering

All procedures in this research were approved by the Research Ethics Committee of Shanghai Ninth People’s Hospital. Primary chondrocytes were isolated from remnant auricular cartilage of microtia patients (average age = 12.7 ± 4.12 years, male : female ≈ 5:4) undergoing the first stage of ear reconstructive surgery. Cartilage was cut into pieces (1-2mm^3^) and digested with 2% Type II Collagenase (Sigma-Aldrich, St. Louis, Mo, USA) for 8 hours at 37°C. Following filtration through a 200-mesh filter net and centrifuging, auricular chondrocytes were separated and cultured in a humidified 37°C and 5% CO_2_ incubator. The DMEM (Dulbecco's Modified Eagle Medium) (Hyclone, Logan City, UT, USA) medium supplemented with 10% fetal bovine serum (FBS, Gibco, USA), 100 U/ml penicillin and 100 μg/ml streptomycin, was renewed every two days. Chondrocytes cultured to the second passage were used in this study as previously described [33]. Chondrocytes were seeded into 6-well plates with 10×10^6^ cells/well density for 8 weeks. The chondrocytes secreted ECM and gradually formed a chondrocyte sheet, which could be detached easily from the plate bottom with a pair of tweezers.

### MicroRNA oligonucleotide transfection and overexpression of MMP16

Self-inactivating lentiviruses (Figure 1A) which express hsa-miR-193b-3p mimics (AACUGGCCCUCAAAGUCCCGCU) and mimics negative control (Mimics-NC) (UUUGUACUACACAAAAGUACUG) miRNAs were purchased from Umibio (Shanghai Co., Ltd., China). After 72 hours, the infection efficiency was detected by immunofluorescence and qPCR (quantitative Polymerase Chain Reaction). MMP16 overexpression plasmids were purchased from RiboBio (Guangzhou Co., Ltd., China). Cells incubated using serum-free DMEM for 24 hours were transfected with plasmids using Lipofectamine 2000 reagent (Invitrogen, Carlsbad, CA, USA) according to the manufacturer’s instructions. Cells without transfection were used as blank controls.

### Evaluation of cell proliferation

Cell proliferation was estimated in 96-well plates using Cell Counting Kit-8 (CCK-8, Dojindo Laboratories, Kumamoto, Japan). The chondrocytes were seeded at 2×10^3^ cells/well supplemented with miR-193b-3p mimics, mimics-NC or DMEM and measured continuously for ten days. A 10 μl CCK-8 reagent was added to each sample and incubated for one hour in a humidified incubator at 37°C with 5% CO_2_; absorbance was then measured at 450 nm by a microplate reader (Thermo Labsystems, Vantaa, Finland). All experiments were performed in triplicate.

### Structural observation of the chondrocyte sheets

The cell sheets sized 1cm^3^ were cleaned three times with PBS (phosphate buffer saline) and fixed with 2.5% glutaraldehyde at 4°C for two hours. After a thorough washing with PBS, the specimens were dehydrated by a gradual concentration change of ethanol and dried by lyophilization. The samples were then coated with platinum and observed by scanning electron microscopy (SEM).

### *In vivo* implantation of the chondrocyte sheets

The *in vitro* cultured chondrocyte sheets were divided into three groups and implanted subcutaneously into nude mice: 1) miR-193b-3p group: chondrocyte sheet cultured with miR-193b-3p mimics; 2) miR-NC group: chondrocyte sheet cultured with miR-193b-3p mimics negative control; 3) Ctrl group: chondrocyte sheet alone (n = 3 for each group, derived from nine nude mice). The samples were harvested at 8 weeks post-implantation for gross view, histological examination, immunohistochemical analysis and immunofluorescence assay.

### Histological and immunohistochemical examinations

The different chondrocyte sheet groups were fixed in 4% paraformaldehyde, dehydrated in a graded ethanol series and then embedded in paraffin. Specimens were stained with Hematoxylin and Eosin (H&E), masson and safranin-O; immunohistological analysis was also carried out. Type II Collagen primary antibodies (rabbit anti human, Servicebio, GB13021, 1:400 dilution) and the secondary antibody (goat anti rabbit, DAKO, K5007, 1:1 dilution) were successively subjected to immunohistological assay. The samples were then observed and photographed under a high-quality microscope.

### Immunofluorescence assay

The different groups of chondrocyte sheets were fixed in 4% paraformaldehyde and embedded in paraffin. Cell sheets were incubated with Type II Collagen primary antibodies (rabbit anti human, Servicebio, GB11021, 1:100 dilution), MMP16 (goat anti human, R&D, AF1785, 1:40 dilution) and a secondary antibody (donkey anti rabbit, Servicebio, GB22403, 1:200 dilution and donkey anti goat, Servicebio, GB21404, 1:300 dilution). The cell nuclei were stained with 4′-6-diamidino-2-phenylindole (DAPI, Servicebio, G1012). The samples were then observed and photographed observed under a high-quality fluorescence microscope.

### Luciferase assay

The DNA sequences of MMP16 3’UTR were amplified by PCR and the seed sequences mutated. The amplified DNA sequences were inserted into the luciferase vectors (RiboBio Co., Ltd., Guangzhou, China) to generate MMP16 3’UTR wild type (wt) or mutated type (mut). The luciferase activity was measured according to the manufacturer’s instructions. Only vectors without cells were used as blank controls. Luminescence was measured and relative luciferase activity calculated. The samples were assayed in triplicate.

### Quantitative real-time polymerase chain reaction (qPCR)

Total RNA for each different group treated with miR-193b-3p mimics, mimics-NC or DMEM was isolated with a Trizol reagent (Life Technologies, Carlsbad, CA, USA) according to the manufacturer’s instructions. Complementary DNA (cDNA) was then synthesized using a PrimeScript 1^st^ Strand cDNA Synthesis kit (Takara, Japan). Quantitative real-time PCR analysis was performed with the Bio-Rad real-time PCR system (Bio-Rad, USA) on the gene expression of COL2A1, MMP16 and miR-193b-3p (Table 1). The relative gene expression levels were calculated by the 2^−ΔΔCt^ method. All experiments were performed in triplicate.

**Table 1:** Primers used in real-time polymerase chain reaction (real-time PCR).

	**Forward primer sequence (5’-3’)**	**Reverse primer sequence (5’-3’)**	**Product size (bp)**
COL2A1	CAG GAT GGG CAG AGG TAT	CGT CTT CAC AGA TTA TGT CGT	109
MMP16	CTA TTC TTC GTC GTG AGA TGT	CCG TCG CTA TTT TCA TAA AC	151
miR-193b-3p	GAA CTG GCC CTC AAA G	TGC GTG TCG TGG AGT C	60
	RT primer (5′-3′): GTCGTATCCAGTGCGTGTCGTGGAGTCGGCAATTGCACTGGATACGACAGCGGGA		
U6	CTT CGG CAG CAC ATA TAC TA	AAC TGG TGT CGT GGA GTC	132
	RT primer (5′-3′): CTCAACTGAATTGCCGACTCCACGACACCAGTTGAGAAAATATG		
β-actin	AAG GTG ACA GCA GTC GGT T	TGT GTG GAC TTG GGA GAG G	195

### Western blot analysis

The protein expression of COL2A1 and MMP16 were examined by western blot analysis. The chondrocytes were treated with miR-193b-3p mimics, mimics-NC, MMP16 expression or DMEM. The specimens were washed twice using PBS and lysed using a RIPA lysis reagent (Thermo Fisher Scientific, Rockford, Illinois, USA). The protein concentrations were determined by bicinchoninic acid (BCA) assay (Pierce Biotechnology, Waltham, Massachusetts, USA). The samples were incubated overnight with primary antibodies including rabbit anti human COL2A1 (Abcam, ab188570, 1:1000 dilution) and goat anti human MMP16 (R&D, AF1785, 1:10000 dilution) at 4°C. The membrane for each antibody was then visualized with horseradish peroxidase (HRP) conjugated secondary antibodies (goat anti-rabbit and donkey anti-goat, Daixuan Bio, 1:500 dilution). The intensities of the protein bands, representative of protein levels, were determined using Quantity One Image software. β-actin was used to normalize target proteins. All experiments were performed in triplicate.

### Statistical analysis

All experiments were performed and analyzed in triplicate. The data are presented as the mean ± SD. Statistical analysis was performed using single-factor analysis of variance (ANOVA) and the Tukey test was used to compare significant differences for multiple groups. A value of *p* < 0.05 was considered to be statistically significant.
